# Elastic Aerogels of Cellulose Nanofibers@Metal–Organic Frameworks for Thermal Insulation and Fire Retardancy

**DOI:** 10.1007/s40820-019-0343-4

**Published:** 2019-12-19

**Authors:** Shengyang Zhou, Varvara Apostolopoulou-Kalkavoura, Marcus Vinícius Tavares da Costa, Lennart Bergström, Maria Strømme, Chao Xu

**Affiliations:** 1 Nanotechnology and Functional Materials, Department of Engineering Sciences, Ångström Laboratory, Uppsala University, 751 21 Uppsala, Sweden; 2 Department of Materials and Environmental Chemistry, Stockholm University, 106 91 Stockholm, Sweden; 3 Applied Mechanics, Department of Engineering Sciences, Ångström Laboratory, Uppsala University, 751 21 Uppsala, Sweden

**Keywords:** Metal–organic frameworks, Nanocellulose, Superelastic aerogel, Thermal insulation, Fire retardancy

## Abstract

Metal–organic frameworks (MOFs) with high microporosity and relatively high thermal stability are potential thermal insulation and flame-retardant materials. However, the difficulties in processing and shaping MOFs have largely hampered their applications in these areas. This study outlines the fabrication of hybrid CNF@MOF aerogels by a stepwise assembly approach involving the coating and cross-linking of cellulose nanofibers (CNFs) with continuous nanolayers of MOFs. The cross-linking gives the aerogels high mechanical strength but superelasticity (80% maximum recoverable strain, high specific compression modulus of ~ 200 MPa cm^3^ g^−1^, and specific stress of ~ 100 MPa cm^3^ g^−1^). The resultant lightweight aerogels have a cellular network structure and hierarchical porosity, which render the aerogels with relatively low thermal conductivity of ~ 40 mW m^−1^ K^−1^. The hydrophobic, thermally stable MOF nanolayers wrapped around the CNFs result in good moisture resistance and fire retardancy. This study demonstrates that MOFs can be used as efficient thermal insulation and flame-retardant materials. It presents a pathway for the design of thermally insulating, superelastic fire-retardant nanocomposites based on MOFs and nanocellulose.

**Electronic supplementary material:**

The online version of this article (10.1007/s40820-019-0343-4) contains supplementary material, which is available to authorized users.

## Highlights

- The study reveals the great potential of metal–organic framework (MOF)-based nanocomposites in thermal insulation and fire retardancy applications.
- A nanoengineering approach was developed to process MOFs into freestanding, mechanically strong, and elastic aerogels, which may boost the fundamental research and practical applications of MOFs in these areas.

**Electronic supplementary material:**

The online version of this article (10.1007/s40820-019-0343-4) contains supplementary material, which is available to authorized users.

## Graphical abstract

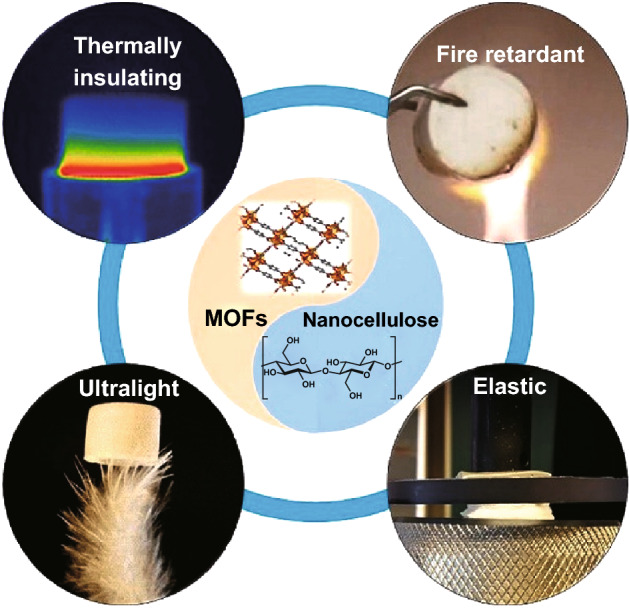

## Introduction

Metal–organic frameworks (MOFs) are an emerging class of porous materials linked by metal-containing nodes and organic ligands via coordination bonds [1, 2]. Taking advantages of their high porosity and diverse structures, MOFs have been received extensive attention on gas storage and separation [3], air purification [4], energy storage [5], drug delivery [6], etc. Meanwhile, there remains great potential to extend their applications. For example, MOFs are theoretically promising thermal insulation materials because of their rich microporosity and hybrid structures [7, 8]. The abundant micropores could suppress gas movement and reduce the mean free path to a few nanometers (versus 75 nm in free space) [9], while the hybrid structures could scatter phonons, thus reducing the thermal conductivity (*λ*) [10]. However, the thermal insulation applications of pure MOFs have rarely been exploited, probably because of the difficulty in shaping and processing of the brittle and insoluble MOF crystals [4, 11].

Apart from their low thermal conductivity, thermal insulation materials are required to be fire-retardant, lightweight, and mechanically resilient or flexible from an application perspective [12–15]. Inorganic thermal insulation materials such as silica aerogels are usually mechanically brittle and difficult to prepare in larger sizes, making them challenging to use in, for example, building or packaging materials [16, 17]. In comparison, polymer-based materials are often flexible and lightweight but suffer from the drawbacks of flammability and poor thermal stability [18]. Organic–inorganic hybridization, a general strategy for the design of functional hybrid materials, provides a feasible design route for hybrid nanocomposites with the potential to merge the advantages of the inorganic and organic components and eliminate their drawbacks [19–23]. For example, Zhao et al. [24] prepared a type of silica-biopolymer aerogels exhibiting significantly improved mechanical properties compared to pure silica aerogels while remaining excellent thermal insulation properties; Kashiwagi et al. [25] reported reduced flammability for poly(methyl methacrylate) after introducing carbon nanotubes. However, the poor compatibility between the organic and inorganic components and their phase separation usually results in uneven dispersion of the components in the composites, which could weaken their thermal insulation performance and simultaneously affect their mechanical properties and lightness [26]. In addition, there remain challenges in the control of the nanostructures and morphology of the composites for optimizing their relevant properties and performances. In this context, we envisioned that nanofabrication of MOFs with appropriate polymer substrates in a controlled manner would offer a feasible approach to process MOFs into functional forms so as to develop the next generation of insulation materials with the integrated properties of good thermal insulation, efficient fire retardancy, low weight, and mechanical resilience.

Cellulose nanofibers (CNFs) have been recently used as building blocks to fabricate nanofibrous composites, which have demonstrated promising properties for various applications [27–33]. There are several advantages in developing CNF-based composites as thermal insulation materials: (1) The naturally abundant and biodegradable CNFs offer a low-cost, sustainable source of materials for manufacturing; (2) CNFs exhibit intrinsically low thermal conductivity; (3) the nanofibrous structure of CNFs results in large interfacial surface areas which act as phonon barriers with potential to hamper heat conduction; (4) CNFs containing organic functional groups on the surface are ideal substrates for modification or hybridization through surface nanoengineering, offering opportunities to overcome the longstanding problems of moisture sensitivity, flammability, and poor mechanical properties associated with CNF-based materials. We have recently developed a range of hybrid nanocomposites based on CNFs for use in energy and environmental applications [12, 34–39]. In this study, we describe the interfacial synthesis and stepwise assembly approach for the design of a hybrid aerogel based on CNFs and an aluminum-based MOF (CNF@Al-MIL-53; CAM). Individual CNFs are coated and further cross-linked with continuously nucleated Al-MIL-53 nanolayers. Because of their special nanostructure, the cross-linked CAM aerogels performed well in thermal insulation and moisture resistance tests, as well as demonstrating superelasticity, high mechanical strength, and fire retardancy.

## Experimental

### Materials

*Cladophora* cellulose powder was ordered from FMC Biopolymer, USA. Aluminum nitrite nonahydrate (Al(NO_3_)_3_·9H_2_O), terephthalic acid, sodium hydroxide (NaOH), and polyvinylpyrrolidone (*M*_w_ = 3.6 × 10^4^ g mol^−1^) were purchased from Sigma-Aldrich without further purification.

### Preparation of CAM Aerogels

CNF@Al-MIL-53 nanofibers were prepared as previously reported, and the details have been provided in Supporting Information [39]. The aqueous suspensions of these CNF@Al-MIL-53 nanofibers (in concentrations of 0.2, 0.5, 1.0, and 2.0 mg mL^−1^) were sealed in homemade copper vessels after ultrasonic treatment in a water bath to remove air bubbles (Fig. S1). The vessels were immersed in liquid nitrogen for 60 min until thermal equilibrium, and the contents were then freeze-dried for 48 h to obtain freestanding aerogels. The aerogels were then infiltrated in a solution of Na_2_BDC (prepared by reacting terephthalic acid with double molar amounts of NaOH in water), and an Al(NO_3_)_3_·9H_2_O solution was added drop-wise. The mixture was gently shaken for 10 h to allow extended growth of Al-MIL-53. The treated aerogels were washed with deionized water several times and then freeze-dried to obtain the cross-linked CAM aerogel.

### Characterizations

Scanning electron microscopy (SEM) images were recorded in a FEG SEM instrument (Zeiss, Leo Gemini 1530) at an accelerating voltage of 3 kV. Transmission electron microscopy (TEM) images were obtained in a TEM instrument (Tecnai, AT02) at an accelerating voltage of 200 kV. X-ray diffraction (XRD) patterns were recorded in a Bruker Focus D8 diffractometer with a Cu-Kα X-ray radiation source (wavelength = 0.154 nm). Fourier-transform infrared spectra were recorded on a Bruker Tensor 27 spectrometer in attenuated total reflection mode. N_2_ sorption isotherms were recorded on a Micromeritics ASAP 2020 surface area and pore size analyzer at 77 K. The samples were degassed at 100 °C under a kinetic vacuum (< 10^−5^ mmHg) for 10 h before the N_2_ sorption measurement. Pore size distributions were calculated from the adsorption isotherms using the density functional theory model. Thermal conductivities were measured using a thermal conductivity testing instrument (Hot Disk 2500S) at room temperature under different humidities. An infrared thermal imaging camera (FLIR, TG165) was used to record the infrared images and the temperature of the aerogels during the high-temperature thermal insulation test. Mechanical properties were measured at room temperature using a Shimadzu Instrument (AGS-X). Heat release rate curves were collected by a cone calorimeter (FTT iCone Mini). Thermogravimetric analysis curves were recorded on a thermogravimetric analyzer (Mettler Toledo, TGA/SDTA851e) under air or N_2_ flow (60 mL min^−1^) between 25 and 800 °C with a heating rate of 10 °C min^−1^.

## Results and Discussions

### Synthesis and Structures of CAM Aerogels

**Fig. 1:**
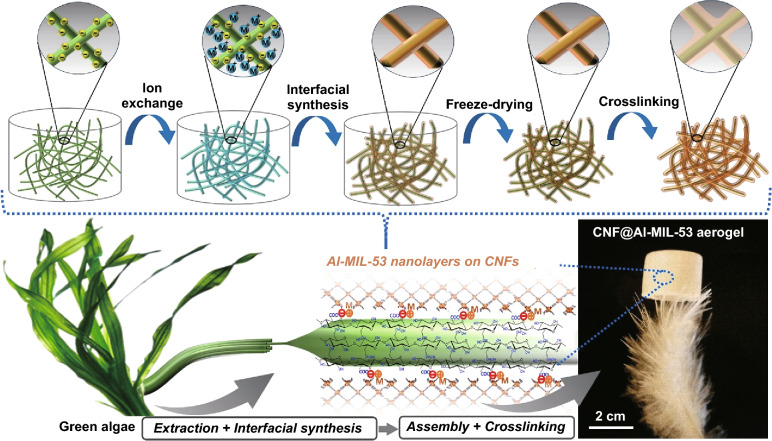
Schematic illustration of the preparation of CNF@Al-MIL-53 (CAM) aerogels through an interfacial synthesis and stepwise assembly approach. Cellulose nanofibers (CNFs) extracted from algae were coated and cross-linked with continuous Al-MIL-53 nanolayers. The resulting freestanding CAM aerogel is ultralight and can stand on the tip of a feather (*ρ *= 0.2 mg cm^−3^)

*Cladophora* cellulose, a type of natural CNFs extracted from green algae, and Al-MIL-53, an aluminum-based MOF, were used to fabricate CAM nanofibers and aerogels. The extensive mesoporosity and mechanical strength of *Cladophora* cellulose were expected to result in aerogels with low thermal conductivity and good mechanical properties (e.g., superelasticity, high compressive modulus), respectively [34]. Similarly, the thermal stability and hydrophobicity of Al-MIL-53 were expected to give the aerogels good fire retardancy and moisture resistance, respectively [40–42]. In addition, the easy synthesis of Al-MIL-53 and the abundance of CNFs offer advantages for large-scale preparation of the hybrid aerogels in a cost-effective manner. Figure 1 provides a schematic summary of the preparation of the CAM aerogel, including (1) interfacial synthesis of CAM hybrid nanofibers, (2) freeze-drying of the CAM nanofibers into the freestanding aerogel, and (3) cross-linking of the aerogel. The original *Cladophora* cellulose was oxidized with TEMPO (2, 2, 6, 6-tetramethylpiperidin-1-yloxyl) to introduce carboxyls on the surface of the CNFs. Next, the carboxylated CNFs underwent ion exchange with Al^3+^ to form the intermediate complex of CNF–COO^−^–Al^3+^. The interfacial synthesis of Al-MIL-53 nanolayers on the CNFs to form the hybrid CAM nanofibers was induced by coordination between the Al^3+^ bonded onto the CNFs and the disodium terephthalate (Na_2_BDC) linker in the presence of polyvinylpyrrolidone as the surfactant and crystallization agent. Freeze-drying the suspension of CAM nanofibers resulted in the corresponding aerogels (Fig. S1). The freestanding aerogel was then immersed into an aqueous solution of Al(NO_3_)_3_∙9H_2_O and Na_2_BDC for the extended nucleation of Al-MIL-53 onto the CAM nanofibers [43–45]. Further freeze-drying of the treated aerogel ultimately formed the target CAM aerogel. It was possible to finely control the density of the CAM aerogels (~ 0.2–3.0 mg cm^−3^) by adjusting the concentration of the suspension of CAM nanofibers (Fig. S2).

**Fig. 2:**
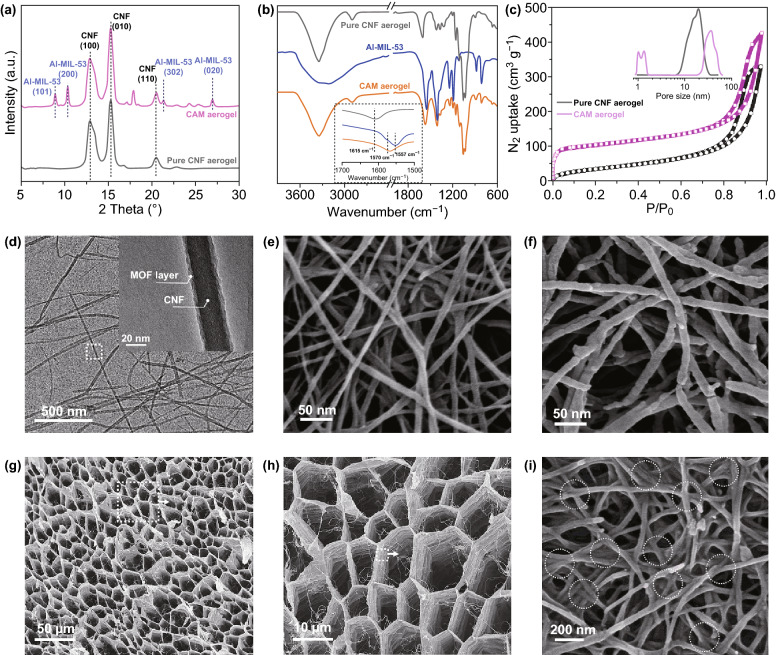
**a** XRD patterns of the pure cellulose nanofibers (CNFs) and CNF@Al-MIL-53 (CAM) aerogels. **b** FTIR spectra of the pure CNF aerogel, pure Al-MIL-53 powder, and CAM aerogel. **c** N_2_ sorption isotherms and pore size distribution of the pure CNF and CAM aerogels. **d** TEM image of CAM nanofibers at different magnifications. High-resolution SEM image of **e** the pure CNFs and **f** the CAM nanofibers. **g–i** SEM images of the CAM aerogel at different magnifications, the circled areas show the joints between the cross-linked nanofibers. MOF = metal–organic framework

The composition of the CAM aerogel was analyzed using powder XRD (Figs. 2a and S3a). The diffraction peaks at 2*θ *= 8.9°, 10.3°, 21.4°, and 26.9° corresponded to the (101), (200), (302), and (020) reflections, respectively, of Al-MIL-53, thus confirming the successful synthesis of Al-MIL-53 [46]. The peaks at 2*θ *= 13.1°, 15.2°, 20.2° corresponded to the (100), (010), (110) reflections, respectively, of the CNFs [38, 39]. Figure 2b compares the Fourier-transform infrared spectra of the carboxylated CNFs, Al-MIL-53, and the CAM aerogel. The IR band at 1615 cm^−1^ for the asymmetric stretching vibration of COO^−^ on the carboxylated CNFs [47] was shifted to 1570 cm^−1^ in the CAM aerogel spectrum, indicating that the carboxyls on the surface of the carboxylated CNFs coordinated with the Al^3+^ to grow the Al-MIL-53 nanolayers. Such shifts were also observed in other metal–carboxylate complexes upon binding to metal ions with higher oxidation states [48]. Likewise, X-ray photoelectron spectroscopy (XPS) studies showed that the binding energies of C 1*s* and O 1*s* in the CAM aerogel were both shifted positively by~ 0.4 eV compared to the values observed in pure CNF (Fig. S4), indicating the change of the coordination environment of COO^−^ in the CNF upon growth of Al-MIL-53 nanolayers. These observations strongly indicated that the Al-MIL-53 nanolayers were chemically grown on the CNFs, rather than being physically bound. The porosity of the CAM aerogel was analyzed by N_2_ sorption measurement (Figs. 2c and S3b). With formation of the microporous Al-MIL-53, the Brunauer–Emmett–Teller (BET) surface area of the CAM (227 m^2^ g^−1^) was much larger than that of the pure CNF aerogel (103 m^2^ g^−1^). Furthermore, pore size distribution analysis revealed the hierarchical porous structure of the CAM aerogel, which contained micropores (~ 1.5 nm) and mesopores (~ 40 nm) originating from the structure of Al-MIL-53 and the inter-fiber stacking, respectively. The mesopores observed in the CAM aerogel were larger than the mesopores in the pure CNF aerogel (~ 20 nm) because assembly of the CAM nanofibers with thicker diameter could form larger inter-fiber voids. Importantly, the content of Al-MIL-53 in the aerogel was increased after the extended nucleation for cross-linking, as confirmed by XRD, N_2_ sorption, and thermogravimetric analysis (TGA) (Figs. S3 and S4c).

The morphology of the CAM nanofibers was analyzed by TEM and SEM. When the CNFs were coated with Al-MIL-53 nanolayers, the hybrid CAM nanofibers, as expected, were larger in diameter (~ 35 nm) than the pure CNFs (~ 20 nm) (Figs. 2d–f and S5a–b). Figure 2d shows the typical core–shell structure of a single CAM nanofiber: An Al-MIL-53 nanolayer is compactly wrapped around the CNF. The high-resolution SEM image of the CAM nanofibers clearly shows their smooth surface, confirming the continuous nucleation of Al-MIL-53 nanolayers onto the CNFs (Fig. 2f). Figures 2g–i show SEM images of the CAM aerogel at different magnifications. Interconnected cellular networks with a pore diameter of ~ 10 μm make up the skeleton of the aerogel (Fig. 2g). This typical cellular architecture is developed during the phase separation of interconnected CAM nanofibers and water in the freeze-drying process [49]. SEM images taken from different directions show similar cellular networks, suggesting the isotropic structure of the aerogel (Fig. S6). The walls of the network are constructed by entangling and weaving of the CAM nanofibers (Fig. 2h). The high-resolution SEM image (Fig. 2i) shows the welded joints between the nanofibers, which indicate the formation of a cross-linked structure in the aerogel. In contrast, this welded nanostructure was not observed in the CAM aerogel before cross-linking and nor was it seen in the pure CNF aerogel (Fig. S7).

### Thermal Insulation and Moisture Resistance Applications

**Fig. 3:**
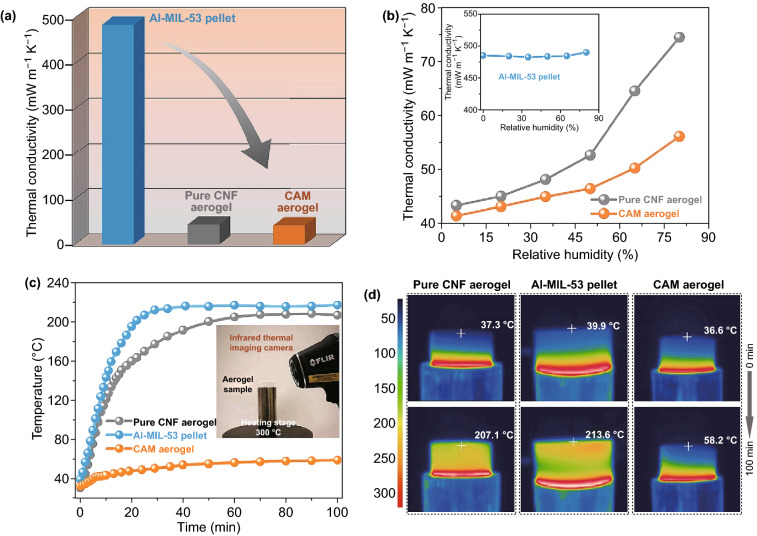
**a** Thermal conductivity of the pure cellulose nanofiber (CNF) aerogel, the pure Al-MIL-53 pellet, and the CNF@Al-MIL-53 (CAM) aerogel at 5% relative humidity and room temperature. **b** Thermal conductivity of the pure CNF aerogel, the pure Al-MIL-53 pellet, and the CAM aerogel as a function of relative humidity at room temperature. **c** Time-dependent temperature profile of the top surface of the pure CNF aerogel, the pure Al-MIL-53 pellet, and the CAM aerogel after placing on a 300 °C heating stage. The inset shows a photograph of the measurement apparatus. **d** Infrared side-view images of the pure CNF aerogel, the pure Al-MIL-53 pellet, and the CAM aerogel, with the temperature of the top surfaces

Given the high porosity and the nanofibrous structure of the CAM aerogel, we expected that it would have relatively low thermal conductivity. Figure 3a compares the thermal conductivity of the pure CNF aerogel, the pure Al-MIL-53 pellet, and the CAM aerogel, measured at 5% relative humidity (RH) and 298 K. As expected, the CNF aerogel with a density of 4.5 mg cm^−3^ had low thermal conductivity (43 mW m^−1^ K^−1^), comparable to values for previously reported cellulose and other polymer-based aerogels [16]. Al-MIL-53 powder samples were pressed into pellets with a density of 1.33 g cm^−3^ for thermal conductivity measurements (Fig. S8). Previous studies suggested that the abundant micropores and the hybrid structures in MOFs could reflect and scatter phonons, thus reducing the lattice thermal conductivity [10]. The thermal conductivity of the Al-MIL-53 pellets was 485 mW m^−1^ K^−1^, which is much lower than the values for bulk inorganic crystalline materials, but higher than those for polymer-based thermal insulators [50, 51]. Remarkably, growing Al-MIL-53 nanolayers on CNFs to form the CAM aerogel (density ≈ 2.6 mg cm^−3^; mass fraction of Al-MIL-53 ≈ 30 wt%) did not increase the thermal conductivity. Instead, the thermal conductivity of the CAM aerogel remained low at 41 mW m^−1^ K^−1^, which is slightly lower than that of the pure CNF aerogel and significantly lower than that of bulk Al-MIL-53.

In general, conduction, radiation, and convection contribute to the thermal conductivity of an aerogel, with the latter two being negligible for isotropic porous materials at room temperature [12, 26, 31, 52, 53]. Hence, conduction, including gas conduction and solid conduction, is the main contributor to the overall thermal conductivity. The gas conductivity *λ*_gas_ of the aerogel can be estimated from Eq. 1 [52]:

1 $$ \lambda_{\text{gas}} = \frac{{\lambda_{g0} \varPi }}{{1 + \frac{{2\beta l_{\text{m}} }}{\delta }}} $$

where $\lambda_{g0}$ is the thermal conductivity of air (~ 25 mW m^−1^ K^−1^), *Π* is the porosity, *β* ≈ 2 for air in aerogels, *l*_m_ is the mean free path of air in specific pores, and *δ* is the average diameter of the pores. The abundant micropores and mesopores in the walls of the CAM aerogel suppress gas movement and reduce the mean free path to a few nanometers (versus 75 nm in free space), thus significantly reducing the gas conductivity within the wall. Meanwhile, the low density of the aerogel results in high porosity (> 99%), which diminishes the contribution of the solid conductivity *λ*_solid_ to the overall thermal conductivity. In addition, the nanofibrous structure and the interfaces between the CNFs and the Al-MIL-53 nanolayers in the CAM aerogel may cause phonon scattering and increase interfacial thermal resistance, which could further reduce the solid conductivity. Therefore, the low thermal conductivity of the CAM aerogel can be attributed to its low density, cellular networks, rich micro-mesoporosity, and hybrid nanofibrous structures.

It is well known that the thermal conductivity of cellulose-based aerogels is highly dependent on the moisture content because of their hygroscopicity, which is one of the drawbacks for their practical application [54]. Figure 3b shows that the thermal conductivity of the pure CNF aerogel significantly increased from 44 at 5% RH to ~ 76 mW m^−1^ K^−1^ (72% increase) at 80% RH. In contrast, the thermal conductivity of the CAM aerogel only increased from 41 at 5% RH to 55 mW m^−1^ K^−1^ (34% increase) at 80% RH, which indicates a significantly lower moisture sensitivity than that of the pure CNF aerogel and of many other CNF-based aerogels [12, 29, 34, 54]. The improved moisture resistance of the CAM aerogel may be associated with the distinct core–shell structure of the hybrid nanofibers, with the hydrophobic Al-MIL-53 nanolayers blocking moisture transportation and reducing moisture uptake by the hydrophilic CNFs at high RH (Figs. S9 and S10).

Furthermore, we developed a proof-of-concept method showing the thermal insulation performance of the CAM aerogel at high temperatures. As shown in Fig. 3c, a 1-cm-thick section of the CAM aerogel was placed on a 300 °C heating stage. The dynamic temperature variation on the top surface of the aerogel during heating was monitored by an infrared thermometer. An Al-MIL-53 pellet and a pure CNF aerogel of the same thickness were similarly tested for comparison. Because of the relatively high thermal conductivity of Al-MIL-53, the temperature on the top surface of the pellet increased rapidly to over 200 °C within 20 min. Although the pure CNF aerogel has low thermal conductivity, it displayed similarly poor thermal insulation performance as the temperature on the top surface reached 200 °C after 50 min. Remarkably, the temperature on the top surface of the CAM aerogel only slightly increased to 50 °C after 30 min and that temperature remained nearly constant after 60 min. After remaining on the heating stage for 100 min, a distinct temperature gradient in a vertical direction through the CAM aerogel was observed from the infrared image (Fig. 3d). Compared to the high temperature of ~ 300 °C for the bottom of the aerogel, the top had a relatively low temperature of 58.2 °C. In contrast, the temperature distributions in both the Al-MIL-53 pellet and the pure CNF aerogel were very homogeneous: The temperature at the top surface finally reached 207.1 and 213.6 °C, respectively. In addition, the bottom of the pure CNF aerogel sample, touching the heating stage, was black after the heat conduction experiment, indicating that the CNF aerogel was partially carbonized (Fig. S11). In comparison, no carbonization was observed for the CAM aerogel. Therefore, the superior thermal insulation performance of the CAM aerogel at high temperatures might also be attributed to its better thermal stability than pure CNFs (Fig. S5c).

### Mechanical Properties of the CAM Aerogel

**Fig. 4:**
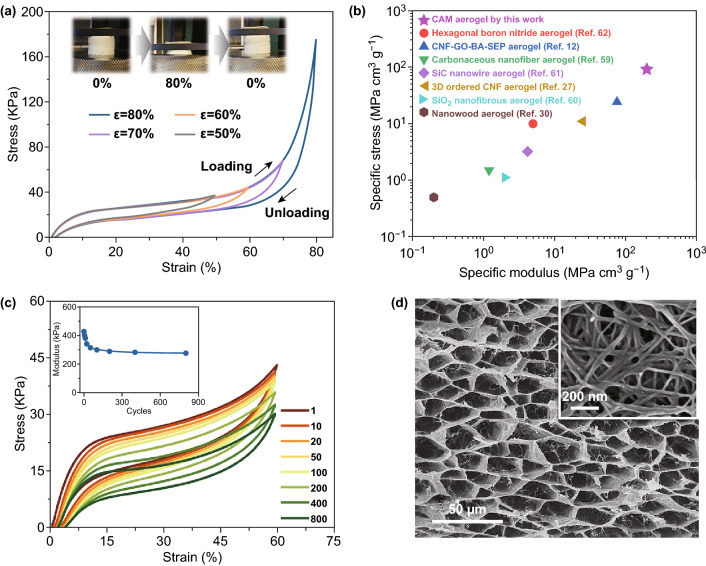
Mechanical properties of the CNF@Al-MIL-53 (CAM) aerogel. **a** Reversible compressive stress–strain curves of the CAM aerogel (*ρ* = 2 mg cm^−3^) using different levels of strain; the insets are photographs of a compression–decompression test cycle. **b** Comparison of the CAM aerogel with other aerogels in terms of specific stress (*σ*/*ρ*) and specific modulus (*E/ρ*). **c** The results of an 800-cycle fatigue test on the CAM aerogel (*ρ* = 2 mg cm^−3^) at a compressive strain of 60%. **d** Scanning electron microscopy images of the CAM aerogel after the 800-cycle compression test

The mechanical properties of the CAM aerogel were assessed using standard compression tests. Unlike the aerogel before cross-linking and the pure CNF aerogel, which were not elastic (Fig. S12), the CAM aerogel with its cross-linked nanostructure had superelastic properties: It recovered its original shape rapidly after releasing the stress (*σ*; Video S1). The compressive stress–strain curves consistently demonstrated that the recoverable compressive strain (*ε*) could reach 80% (Fig. 4a). The strain gradually decreased to zero during the release of the stress, and the hysteresis loop area was relatively small (Figs. 4a and S13b–d). In addition, the stress–strain loops were almost identical when the compression rate was increased from 20 to 800 mm min^−1^. The fast recovery rate of the CAM aerogel indicates its suitability for application in stress sensors, shape-memory materials, etc (Fig. S13).

The stress–strain curves of the CAM aerogel showed three distinct deformation stages, which is a characteristic of aerogels with open cellular networks [55–58]: (1) The stress increased linearly at low strains (*ε* < 10%), suggesting the elastic deformation of the aerogel caused by cell walls bending; (2) a stress plateau was observed at 10% < *ε* < 60%, reflecting the deformation of the cellular macropores; and (3) the stress increased steeply in the densification region for *ε* > 60%, resulting from close contact with and further compression of the cell walls. The mechanical strength of the CAM aerogel was significantly improved over that of the pure CNF aerogel. The compressive modulus, yield strength, and ultimate stress of the CAM aerogel were 430, 22, and 180 kPa, respectively; these are much higher than the corresponding values for the pure CNF aerogel and comparable with those of carbon, ceramic, and polymer aerogels (Table S1). More significantly, given the low density of the CAM aerogel, the specific modulus (*E/ρ*) and ultimate stress (*σ/ρ*) reached ~ 200 MPa cm^3^ g^−1^ and ~ 100 MPa cm^3^ g^−1^, respectively; these are significantly higher than the corresponding values of previously reported CNF-based aerogels [14, 27, 30] and even of some inorganic aerogels based on boron nitride nanosheets, carbon nanofibers, SiC nanofibers, and SiO_2_ nanofibers (Fig. 4b) [59–62]. Another important finding was that the relative compressive modulus (*E/E*_s_) displayed a roughly linear dependence on the relative density (*ρ/ρ*_s_) for the CAM aerogels (*E/E*_s_~(*ρ/ρ*_s_)^α^, *α* ≈ 1) (Fig. S14), which implies that the aerogels can effectively spread and equilibrate the external stress through the overall framework, thanks to their homogeneous and cross-linked network structures [27]. In contrast, the dependence of the modulus on the density of the pure CNF aerogels and traditional inorganic aerogels meant that the values for α in the equation *E/E*_s_~(*ρ/ρ*_s_)^α^ were higher (*α *≈ 2 and 3, respectively) [63]. Obviously, the high specific modulus and the cross-linked network structure of the CAM aerogels are of great importance for their use as high performance, lightweight structural materials with mechanical strength and stability.

The compression stability of the CAM aerogel was evaluated in cyclic compression tests under high levels of strain (60%) and a loading rate of 100 mm min^−1^ (Fig. 4c). The stress–strain loops almost overlapped during the 100 cycles tested. Of note, the aerogel shrank only 8.3% in volume (plastic deformation) and retained over 70% of the original modulus after 800 loading–unloading cycles, indicating its high mechanical stability. Moreover, we compared the SEM images of the aerogel before and after 800 cycles of compression (Fig. 4d). Surprisingly, the cellular networks and cross-linked nanostructure were almost unchanged after compression, indicating the high structural stability of the aerogel. The superelasticity and the high mechanical strength of the CAM aerogel can thus be explained by the stable cellular network and the highly cross-linked nanostructure effectively preventing collapse of the walls during compressive deformation.

### Fire Retardancy Applications

**Fig. 5:**
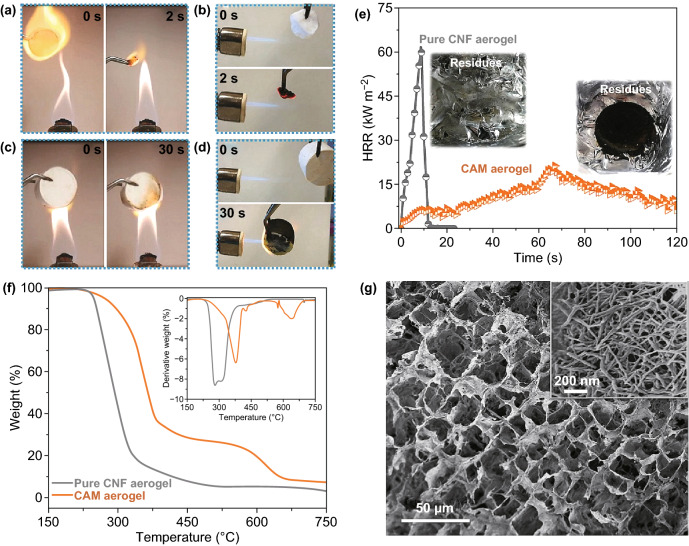
Burning tests of **a** the pure cellulose nanofiber (CNF) aerogel in the flame of an alcohol lamp (~ 500 °C), **b** the pure CNF aerogel in the flame of a butane blowtorch (~ 1300 °C), **c** CNF@Al-MIL-53 (CAM) aerogel in the flame of an alcohol lamp, and **d** CAM aerogel in the flame of a butane blowtorch. **e** Heat release rate (HRR) curves of the pure CNF and CAM aerogels recorded during the cone calorimetry test. The insets show the residues after the measurements. **f** TGA analysis curves of the pure CNF and CAM aerogels under air flow. **g** High-resolution SEM images of CAM aerogel after the combustion experiment in the flame of a butane blowtorch

The use of organic thermal insulators has been limited by their flammability. As expected, the pure CNF aerogel can be easily ignited and burned within 3 s in the flame of an alcohol lamp (~ 500 °C) (Fig. 5a). Although the pure Al-MIL-53 pellet was nonflammable, the blended CNF-Al-MIL-53 aerogel composed of CNFs and Al-MIL-53 nanoparticles shrank rapidly upon exposure to the flame before transforming to black ash (Fig. S15). This can be explained by the dispersed Al-MIL-53 nanoparticles not forming interconnected networks with the CNFs and thus not protecting them. In obvious contrast to the highly flammable CNF and CNF-Al-MIL-53 aerogels, the CAM aerogel did not ignite and the flame did not self-propagate (Figs. 5c and S16, Video S2). There was only slight volume contraction after exposing the aerogel to the flame of the alcohol lamp for 30 s. More significantly, the CAM aerogel displayed excellent fire retardancy and remained intact even under the flame of a butane blowtorch (~ 1300 °C), while the pure CNF aerogel quickly burned, leaving no residue (Fig. 5b, d, and Video S3). In addition, the fire-retardant properties of the aerogels were quantitatively evaluated in a cone calorimetry study. The heat release rate (HRR) curves are presented in Figs. 5e and S14b. Consistent with the results of the combustion experiments, the flammable pure CNF and CNF-Al-MIL-53 aerogels released substantial heat over a short period (~ 10 s) with peak HRR (pkHRR) values of ~ 60 and 52 kW m^−2^, respectively. In contrast, the combustion behavior of the CAM aerogel was significantly different, with a much lower pkHRR value of ~ 19 kW m^−2^ at a delayed peak time of 65 s.

To better understand the good fire retardancy of the CAM aerogel, we studied its thermal stability in air using TGA (Fig. 5f). Compared to the decomposition temperatures (*T*_d_) of the pure CNF aerogel at ~ 275 °C, the first *T*_d_ observed in the CAM aerogel arising from the thermal degradation of CNFs was significantly increased to ~ 375 °C. This result evidences the thermal protective effect of the Al-MIL-53 nanolayers on the CNFs. The second *T*_d_ at ~ 640 °C indicates the thermal decomposition of Al-MIL-53 to form aluminum oxide, as confirmed by the XRD studies (Fig. S17). The microstructure of the CAM aerogel after combustion (under the flame of a butane blowtorch for 30 s) was analyzed by SEM. It was apparent that the aerogel maintained the cellular network and cross-linked nanofibrous structures (Figs. 5g and S17). In contrast, no nanofibrous structure was observed in the residues of the CNF-Al-MIL-53 aerogel (Fig. S15), confirming that the CNFs were totally burned. These findings strongly support the notion that the special core–shell structure of the CAM nanofibers plays a key role in the fire retardancy of the aerogel. It is suggested that the Al-MIL-53 nanolayers and the thermally decomposed aluminum oxide efficiently protected the wrapped CNFs from ignition upon exposure to flames (Fig. S17) [64–66]. In addition, the stable cellular network structures and the abundant hierarchical pores in the thermally insulating CAM aerogel could serve as intrinsic barriers to prevent heat transfer from the flame to the interior, which could also explain the good fire retardancy of the aerogel.

## Conclusions

In summary, we have designed a novel hybrid CAM aerogel using a stepwise assembly approach involving the coating and cross-linking of CNFs with MOF nanolayers. First, Al-MIL-53 nanolayers were synthesized on the surface of CNFs to form integrated nanofibers with a distinct core–shell nanostructure. Next, extended growth of Al-MIL-53 on the hybrid nanofibers formed freestanding cross-linked, nanofibrous CAM aerogels. Because of their high porosity, cellular networks, and nanofibrous structure, the obtained aerogels demonstrated relatively low thermal conductivity (~ 40 mW m^−1^ K^−1^), suggesting the potential for high-temperature insulation applications. The hydrophobic, thermally stable Al-MIL-53 nanolayers coated on the CNFs provided not only high moisture resistance but also good fire retardancy (under a butane blowtorch at ~ 1300 °C) for the CAM aerogels, paving the way for a solution to the longstanding challenges of moisture sensitivity and flammability faced by various biopolymer aerogels. Moreover, the CAM aerogels with their cross-linked structures showed superelasticity (80%) and high specific mechanical strength (*E/ρ*: ~ 200 MPa cm^3^ g^−1^; *σ/ρ*: ~ 100 MPa cm^3^ g^−1^). This study opens up unprecedented possibilities for developing MOF-based nanocomposites for thermal insulation and fire retardancy applications. The newly developed aerogels based on MOFs and sustainable celluloses (and perhaps other biopolymers) may find application in energy-efficient buildings, structural materials, packaging and storage of food, and pharmaceuticals.

## Electronic supplementary material

Below is the link to the electronic supplementary material.

Supplementary material 1 (PDF 1899 kb)

Supplementary material 2 (MP4 716 kb)

Supplementary material 3 (MP4 2720 kb)

Supplementary material 4 (MP4 890 kb)
