# Supplementary material for: Elastic Aerogels of Cellulose Nanofibers@Metal–Organic Frameworks for Thermal Insulation and Fire Retardancy
Source: Nanomicro Lett. 2019 Dec 19;12:9. doi: 10.1007/s40820-019-0343-4 (PMC7770683; doi:10.1007/s40820-019-0343-4)
Supplement: Supplementary file 1 — Supplementary material 1 (PDF 1899 kb) [file 40820_2019_343_MOESM1_ESM.pdf]

Supporting Information for

Elastic Aerogels of Cellulose Nanofibers@Metal-Organic Framework for Thermal Insulation and Fire Retardancy

Shengyang Zhou[1], Varvara Apostolopoulou-Kalkavoura[2], Marcus Vinícius Tavares da Costa[3], Lennart Bergström[2], Maria Strømme[1,*], Chao Xu[1,*]

[1]Nanotechnology and Functional Materials, Department of Engineering Sciences, Ångström Laboratory, Uppsala University, 751 21 Uppsala, Sweden

[2]Department of Materials and Environmental Chemistry, Stockholm University, 10691 Stockholm, Sweden

[3]Applied Mechanics, Department of Engineering Sciences, Ångström Laboratory, Uppsala University, 751 21 Uppsala, Sweden

*Corresponding authors. E-mail: maria.stromme@angstrom.uu.se (M. Strømme); chao.xu@angstrom.uu.se (C. Xu)

S1 Thermal Conductivity Measurement

The thermal conductivity (λ, mW mK^{-1}) of the samples was measured in isotropic mode using the TPS 2500 S Hot Disk Thermal Constants Analyzer as it was described before by Apostolopoulou-Kalkavoura et al. [S1]. The TPS sensor (6.4 mm in radius) was placed between two identical samples and in order to be sure that the thermal contact between the sensor and the samples was good a small weight was added on the top of the samples [S3, S4]. The samples were then placed in a customized cell [S1] connected with a P2 Cellkraft humidifier[2] allowing careful control of the relative humidity (RH). To control the temperature (T), the measurement cell was immersed in a silicon oil bath. The samples were tested between 5 and 80% RH at T = 295 K. To ensure stable conditions, the samples were conditioned for at least 2 h at the targeted T and RH and then 5 measurements with 15 min interval were performed for each sample. The heating power was 10-20 mW and the measurement time was 10-20 s for all the CNF-based aerogels tested and 80 mW and 40 s respectively for the pure MOF pellet.

S2 Supplementary Figures and Table

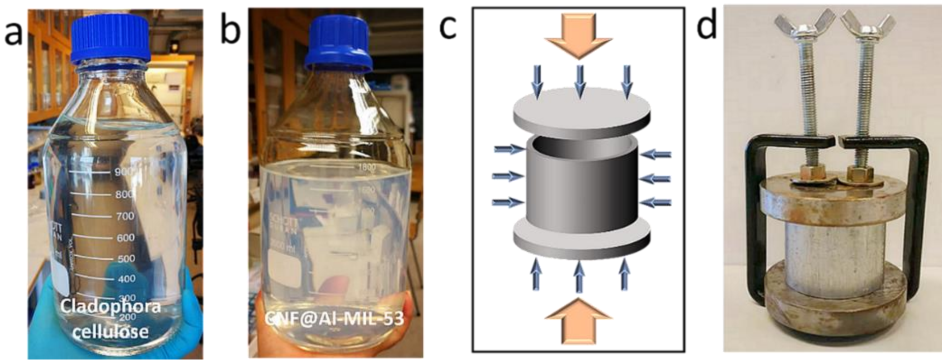

Fig. S1 Photograph of aqueous suspensions of **a** pure *Cladophora* CNFs after TEMPO oxidation and **b** CNF@Al-MIL-53 hybrid nanofibers. **c** chematic diagram and **d** hotograph of the apparatus for the preparation of aerogels by rapid freeze-drying

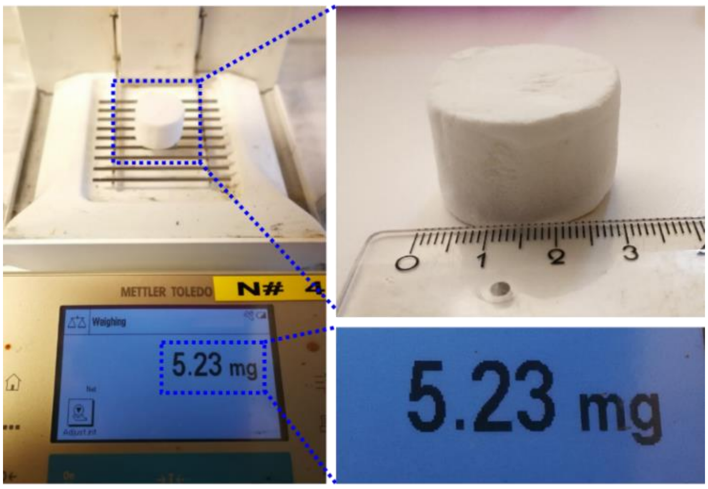

Fig. S2 Photograph of a CNF@Al-MIL-53 aerogel with the density of ~1.0 mg cm^{-3}

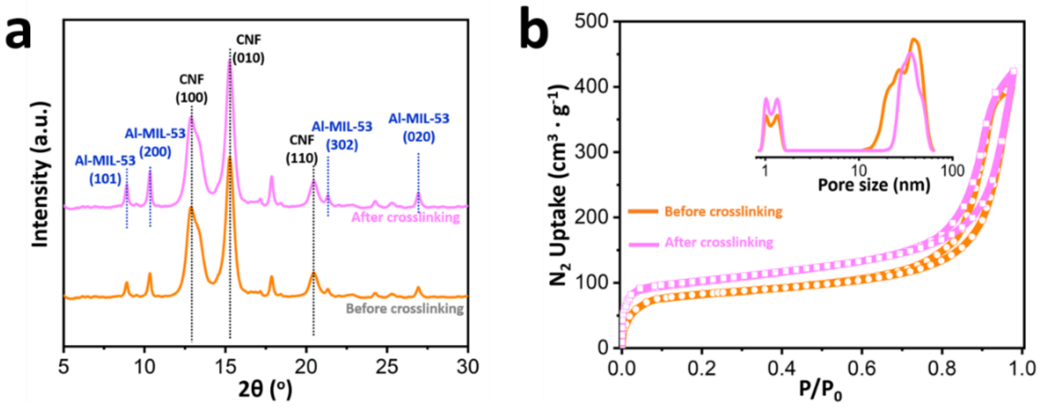

Fig. S3 **a** XRD patterns, and **b** N_2 sorption isotherms and pore size distribution of CNF@Al-MIL-53 aerogels before and after crosslinking

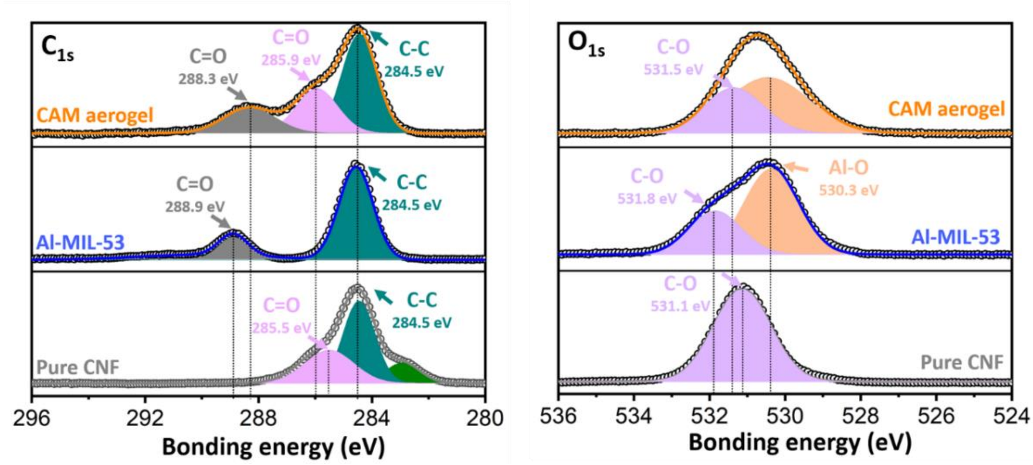

Fig. S4 High-resolution C 1s and O 1s X-ray photoelectron spectra of pure CNF, pure Al-MIL-53, and CAM aerogel

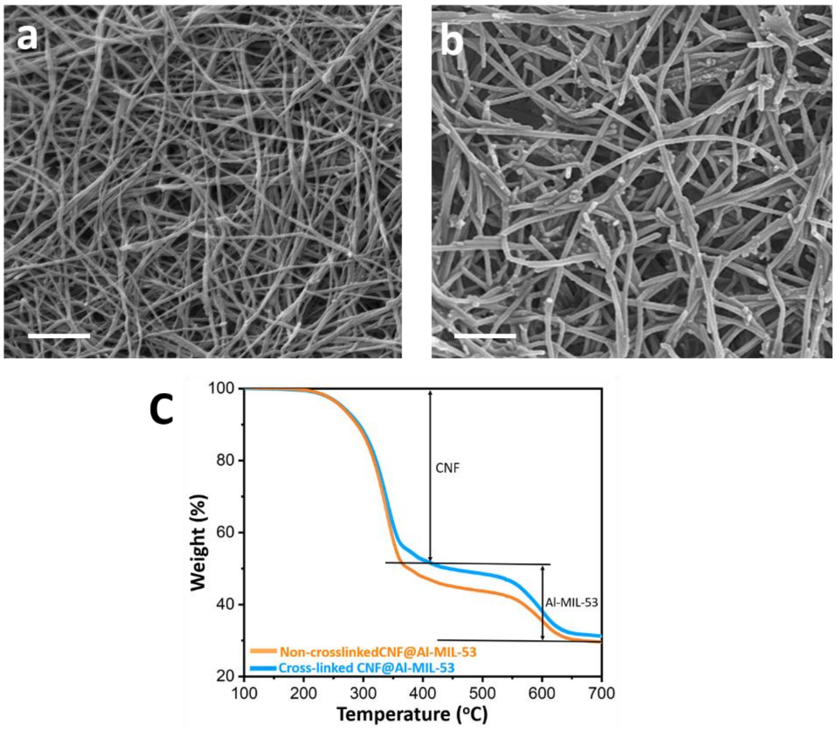

Fig. S5 SEM image of **a** pure *Cladophora* cellulose after the TEMPO oxidation and **b** CNF@Al-MIL-53 hybrid nanofibers, the scale bar is 300 nm. **c** TGA curves of the CNF@Al-MIL-53 aerogel before and after crosslinking under a N_2 flow

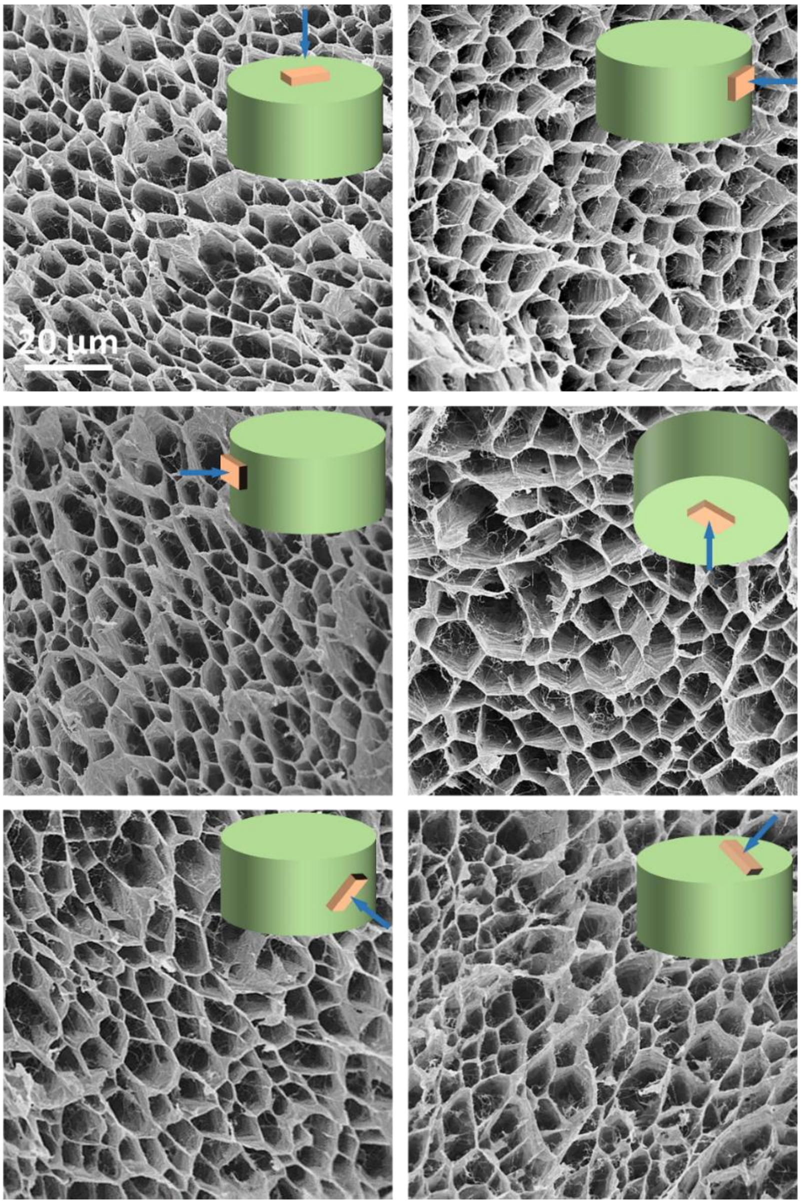

Fig. S6 SEM image of the CNF@Al-MIL-53 aerogel recorded from different directions

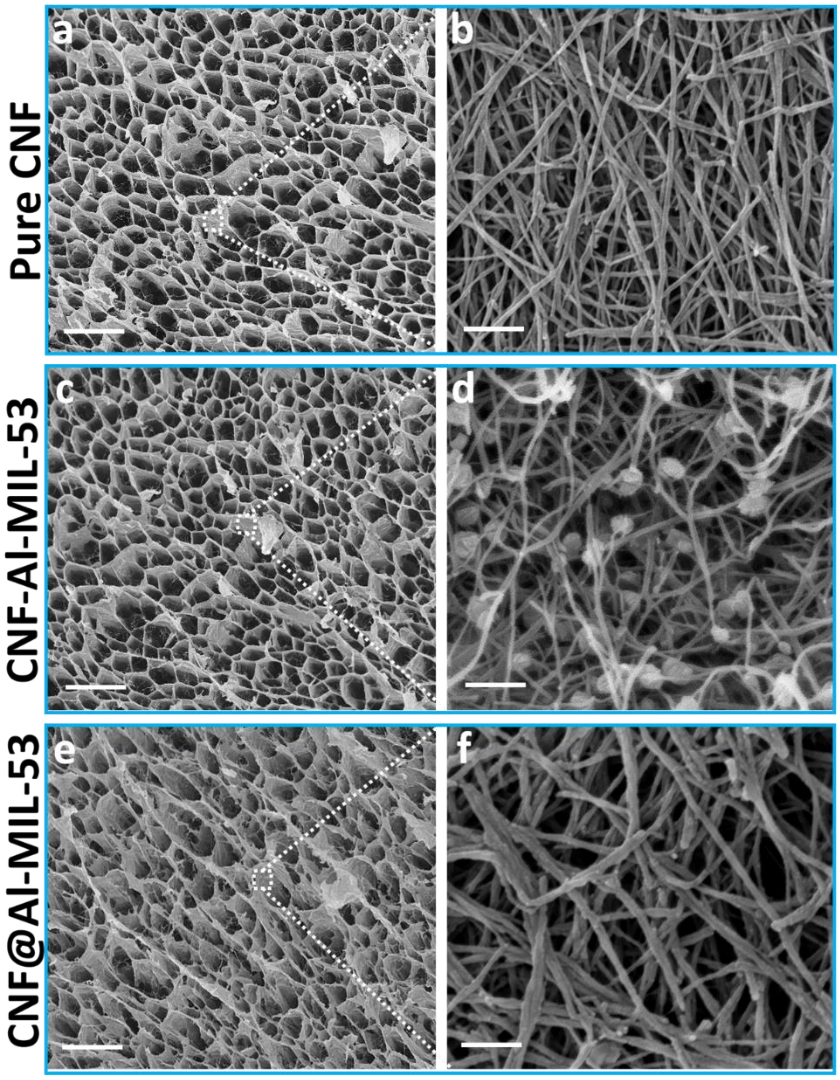

Fig. S7 **a**, **b** SEM images of the pure CNF aerogels; **c, d** SEM images of CNF-Al-MIL-53 aerogels by direct mixing CNFs and Al-MIL-53 nanoparticles; **e, f** SEM images of the CNF@Al-MIL-53 aerogel before crosslinking. Scale bar: 30 µm (**a, c, e**) and 200 nm (**b, d, f**)

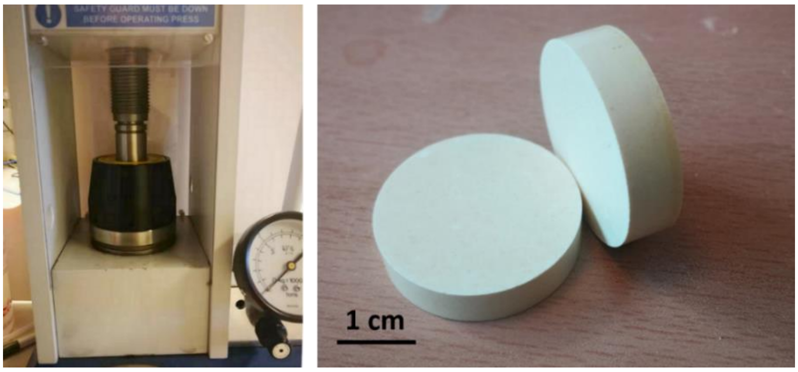

Fig. S8 **a** Equipment for pressing the powder of Al-MIL-53 into pellet. **b** Photograph of the pure Al-MIL-53 pellets pressed under 300 MPa

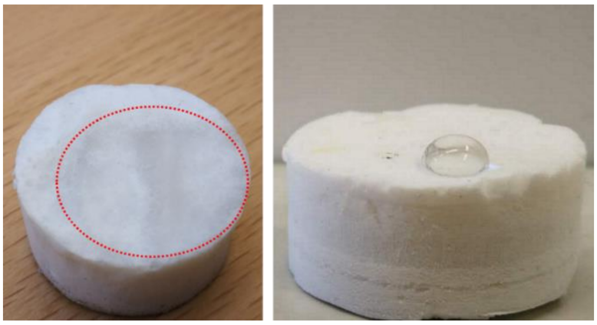

Fig. S9 Photograph of the water droplet on the surface of **a** the pure CNF and **b** CNF@Al-MIL-53 aerogel showing that the pure CNF aerogel is hydrophilic and the CNF@Al-MIL-53 aerogel is hydrophobic

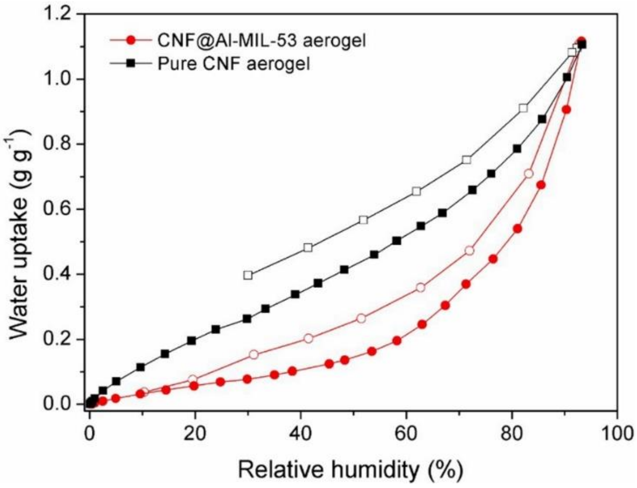

Fig. S10 Water vapor sorption isotherms of pure CNF and CNF@Al-MIL-53 aerogels at 293 K. The water vapor adsorption uptake for the CNF@Al-MIL-53 aerogel is much lower than the pure CNF aerogel at RH = 0−80%, indicating that the AL-MIL-53 nanolayer coated on CNF could block water vapor transportation and result somewhat hydrophobicity for the CNF@Al-MIL-53 aerogel

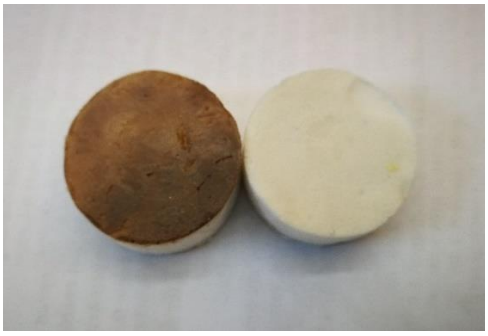

Fig. S11 Photograph of the pure CNF aerogel (left), and CNF@Al-MIL-53 aerogel (right) after the high-temperature thermal insulation test. The surface of the aerogels was put on the heating stage (300 °C) for 100 min

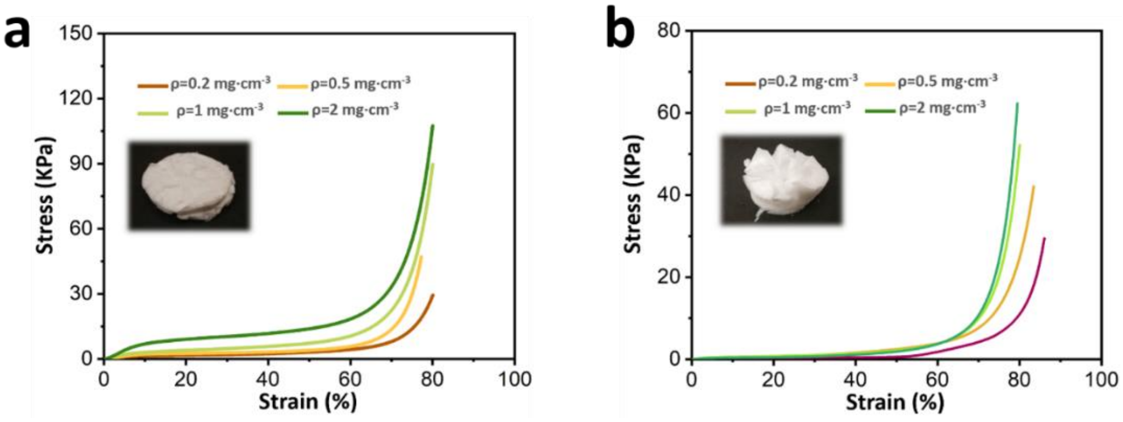

Fig. S12 Compressive stress-strain curves of **a** pure CNF and **b** non-crosslinked CNF@Al-MIL-53 aerogels with different densities. The results indicated that the pure CNF aerogel and non-crosslinked CNF@Al-MIL-53 aerogel are inelastic

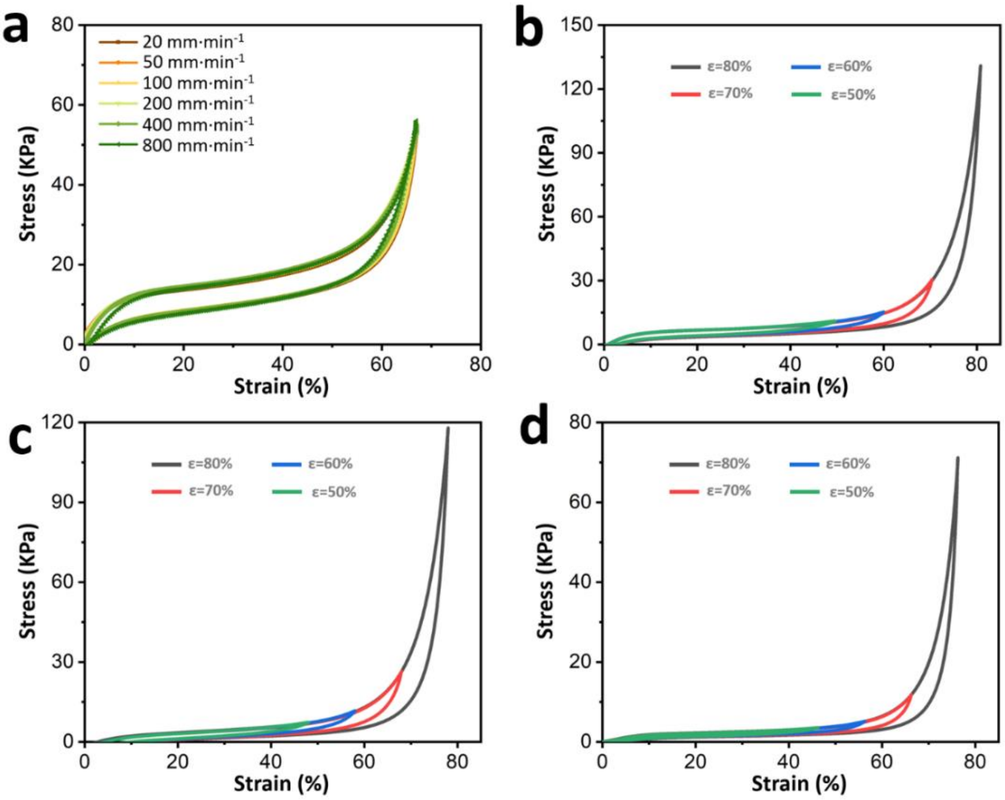

Fig. S13 a Reversible compressive stress-strain curves of the CNF@Al-MIL-53 aerogel (ρ = 2 mg cm^{-3}) with different compression rates. Reversible compressive stress-strain curves of the CNF@Al-MIL-53 aerogel with different density under a loading rate of 100 mm min^{-1}: **b** 1 mg cm^{-3}, **c** 0.5 mg cm^{-3}, **d** 0.2 mg cm^{-3}

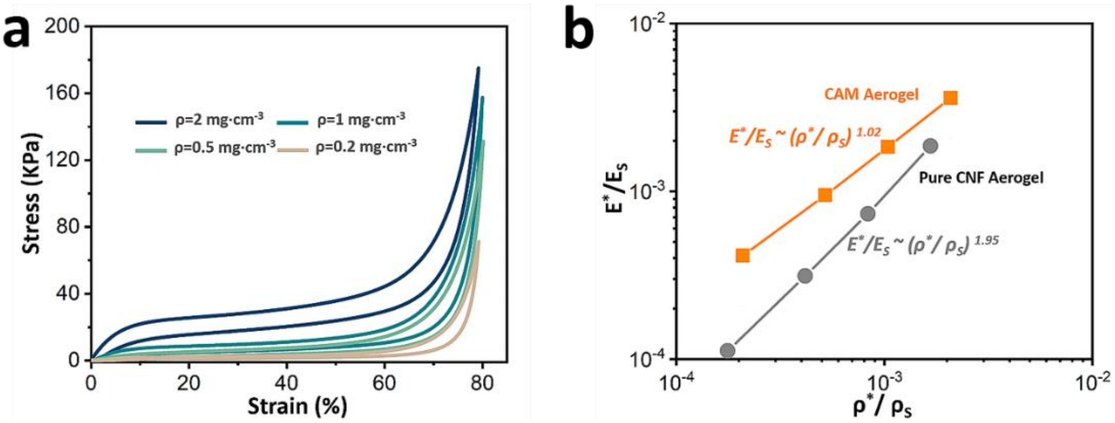

Fig. S14 a Reversible compressive stress-strain curves of the CNF@Al-MIL-53 aerogels with different densities. **b** The relative modulus of the pure CNF and CNF@Al-MIL-53 aerogels at various relative densities

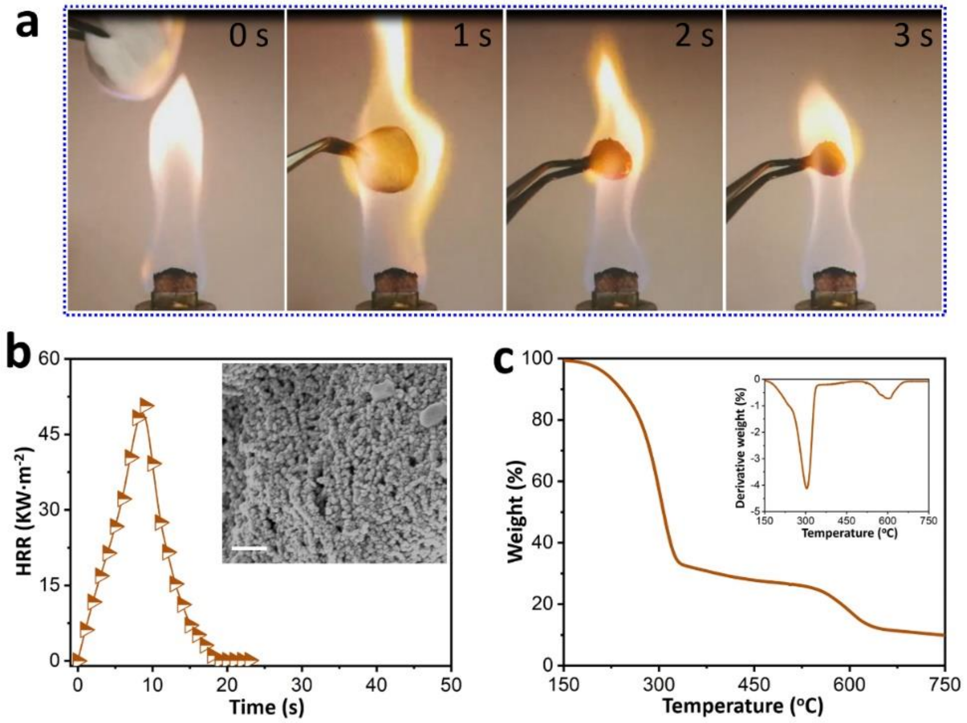

Fig. S15 a Burning test of the CNF-Al-MIL-53 aerogel (prepared by direct mixing CNFs and Al-MIL-53 nanoparticles) in the flame of alcohol lamp (~500 °C. **b** Heat release rate curves of the CNF-Al-MIL-53 aerogel, the inset showing its SEM image after burning, scale bar: 200 nm. **c** TGA curve of the CNF-Al-MIL-53 aerogel under a N_2 atmosphere. The first derivative plot of the TGA curve shows that the decomposition temperatures for CNF and Al-MIL-53 are 300 and 600 °C, respectively, which are lower than the values observed in the CNF@Al-MIL-53aerogel. These results indicates that blending Al-MIL-53 nanoparticles with CNF could not increase the thermal stability and flame retardancy for the aerogel

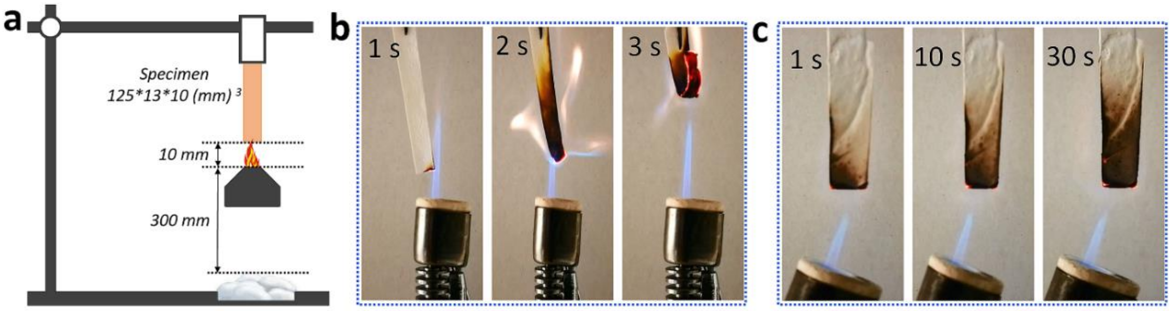

Fig. S16 a Illustration of vertical burning test (UL94). Photos of **b** pure CNF and **c** CNF@Al-MIL-53 aerogel during the burning test at different time points

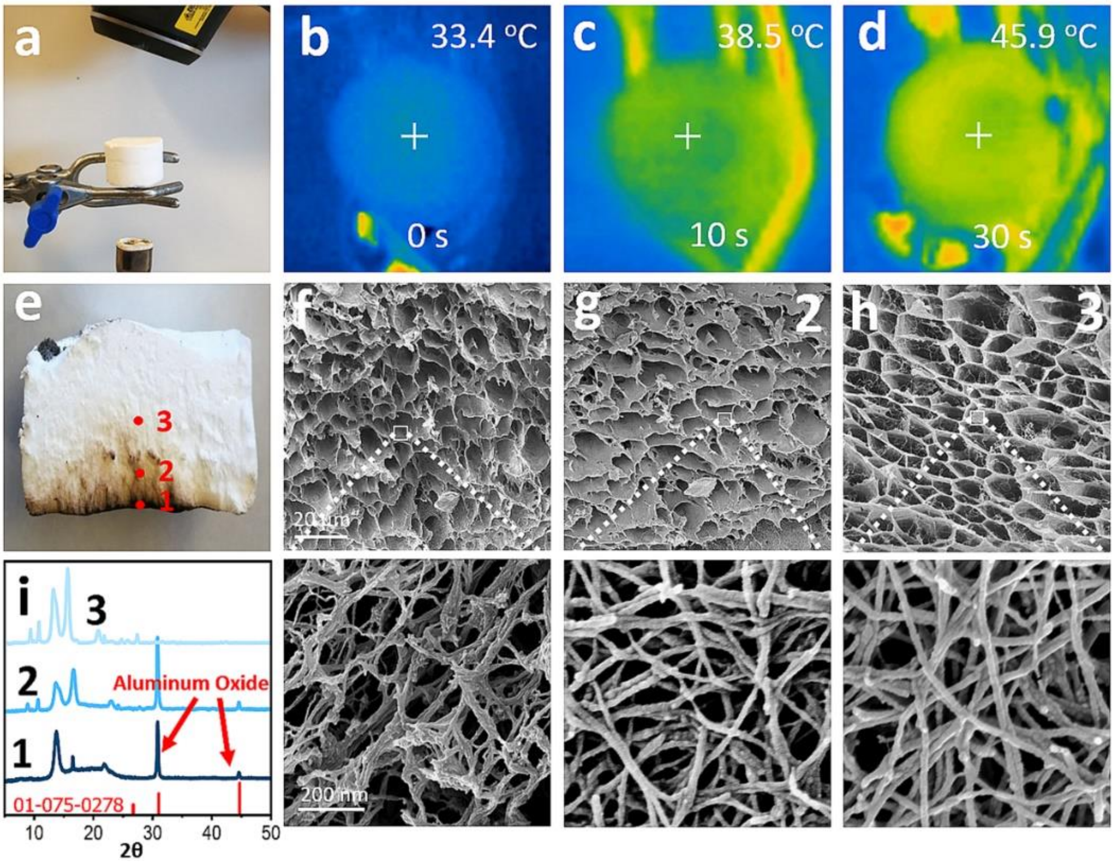

Fig. S17 a The set-up for the burning test. The bottom of the CNF@Al-MIL-53 aerogel was burned by the flame of butane torch and the temperature at the top surface was monitored by an infrared camera. **b-d** Infrared images of the aerogel from a top view and the temperature variation at the top surface during the burning test. **e** A profile photo of the CNF@Al-MIL-53 aerogel after placing in the flame for 30 s. **f-h** SEM images and **i** XRD patterns of the CNF@Al-MIL-53 aerogel taken from different points after the burning test

Table S1 Comparison of the mechanical property of pure CNF and CNF@Al-MIL-53 aerogels with reported aerogels

Aerogels	Elastic Modulus (kPa)	Yield Stress (kPa)	Ultimate Stress (kPa)
**Pure CNF**	190	7	105
**Non-crosslinked CAM**	23	-	63
**Crosslinked CAM**	420	22	180
**Carbon Nanofibrous** (Adv. Mater. 2019, 1900651)	100	5	40
**Carbon** (Angew. Chem. 2013, 125, 2997 –3001)	15	-	60
**Polymethylsilsesquioxane** (ACS Appl. Mater. Interfaces 2014, 6, 9466-9471)	300	-	200
**Polyvinylpolydimethylsiloxane** (Angew. Chem. Int. Ed. 2018, 57, 9722 –9727)	-	2	400
**Ceramic Nanofiber** (Sci. Adv. 2017, 3, e1603170)	20	-	5
**Ceramic** (Science 2019, 363, 723–727)	24	-	50

Supplementary References

[S1] V. Apostolopoulou-Kalkavoura, K. Gordeyeva, N. Lavoine, L. Bergström, Thermal conductivity of hygroscopic foams based on cellulose nanofibrils and a nonionic polyoxamer. Cellulose **25,** 1117–1126 (2018). https://doi.org/10.1007/s10570-017-1633-y

[S2]A. Ocklind, Humidifier P-series. *Cellkraft* 1–3 (2016). Available at: http://www.cellkraft.se/downloads/P-Series.pdf

[S3]C. Dixon, M.R. Strong, S.M. Zhang, Transient plane source technique for measuring thermal properties of silicone materials used in electronic assemblies. Int. J. Microcircuits Electron. Packag. **23,** 494–500 (2000).

[S4]S.E. Gustafsson, Transient plane source techniques for thermal conductivity and thermal diffusivity measurements of solid materials. Rev. Sci. Instrum. **62,** 797–804 (1991). https://doi.org/10.1063/1.1142087
